# Breathlessness and presentation to the emergency department: a survey and clinical record review

**DOI:** 10.1186/s12890-017-0396-4

**Published:** 2017-03-20

**Authors:** Ann Hutchinson, Alistair Pickering, Paul Williams, J. Martin Bland, Miriam J. Johnson

**Affiliations:** 1 Hull York Medical School, University of Hull, Hull, HU6 7RX UK; 2 HEYHT, Hull, UK; 3 University of York, York, UK

**Keywords:** Epidemiology, Breathlessness

## Abstract

**Background:**

Breathlessness is a frequently occurring symptom of cardiorespiratory conditions and is a common cause of emergency department presentation. The aim of this study was to estimate the prevalence of acute-on-chronic breathlessness as a cause for presentation to the major emergencies area of the emergency department.

**Methods:**

A prospective patient self-report survey and clinical record review of consecutive attendees to the major emergencies area of the emergency department in a single tertiary hospital between 12/5/14 and 29/5/14 was conducted. Eligible patients were clinically stable and had mental capacity to provide data.

**Results:**

There were 2,041 presentations during the study period, of whom 1,345 (66%) were eligible. There was a 90% survey response rate (1,212/1,345); 424/1,212 (35%) self-reported breathlessness most days over the past month of whom 245 gave breathlessness as a reason for this presentation. Therefore, the prevalence of acute-on-chronic breathlessness as a reason to present to the major emergencies area was 20.2% (245/1,212, 95% CI 17.9% to 22.5%). During this period there were 4,692 major *and* minor presentations; breathlessness was therefore a cause of *at least* 5.2% (245/4,692, 95% CI 4.6 to 5.9%) of all emergency department presentations.

**Conclusions:**

This study found that one in five ambulance presentations to the ED were due to acute-on-chronic breathlessness. Most patients had non-malignant underlying conditions, had experienced considerable breathlessness for an extended period, had discussed breathlessness with their GP and presented out of daytime hours. Others were often involved in their decision to present. This represents clinically significant burden for patients, their family carers and the emergency health services.

## Background

Breathlessness, medically known as dyspnoea, is a common presenting symptom in the emergency department (ED). Data from the 2007 Unites States National Hospital Ambulatory Medical Care Survey show that “shortness of breath” and/or “dyspnea” were amongst the top ten principal reasons for adult presentation to the ED; comprising 3.2% that year [1]. An estimate from the more recent 2013 survey is that shortness of breath accounted for 3.0% of all adult ED presentations [2]. Other estimates of prevalence of breathlessness as a primary reason for presentation range between 2.7% and 9% depending on the breathlessness measure used and population [3–5].

Breathlessness is a feature of cardiorespiratory conditions [6] and its intensity on arrival at the ED predicts hospital admission as a post-presentation destination [7]. One clinical record review showed that a quarter of people admitted to hospital from the ED were those presenting with breathlessness [8].

Breathlessness is also associated with return presentation to the ED [9] suggesting that management of the breathlessness remains challenging. These studies did not differentiate between acute and acute-on-chronic breathlessness. However those with acute-on-chronic breathlessness are a group which may include individuals, such as those where anxiety plays a significant role, for whom targeted crisis management plans [10] may prevent avoidable re-attendance and hospital admission [11]. Furthermore, previous studies have relied on clinical documentation rather than on patient self-report, risking an underestimate of the prevalence of breathlessness; a subjective sensation. Discrepancy between the assessment of breathlessness by doctors and patients has been noted [12]. Clinicians also vary in their beliefs about breathlessness, its impact on the patient and on strategies for its management [13].

The primary aim was to estimate the prevalence of patient-reported acute-on-chronic breathlessness as a reason for presentation to the major emergencies area (“majors”) of the ED. Secondary objectives were to describe patient clinical and demographic characteristics and the circumstances regarding the decision to present and outcomes of presentation.

Our hypothesis was that acute-on-chronic breathlessness would be a reason for presentation in at least 3.2% of presentations.

## Methods

### Study design

A prospective consecutive patient-report survey and clinical record review was conducted in a single, tertiary hospital. The survey was developed through a process of extensive peer review and patient feedback.

Chronic breathlessness was defined as self-report of experiencing shortness of breath “most days in the last month”. The patient self-report survey measured the prevalence of acute-on-chronic breathlessness as a cause for presentation to the majors area of the ED. The survey was administered by clinical staff either after the patient had been through triage and was waiting to be seen by a doctor or once they had stabilised in the resuscitation area. The first page of the survey had a brief introduction to the study, stating that it was to understand problems of people coming to the emergency department and also questions on age, postcode and gender, as well as the key question whether they experienced of chronic breathlessness or not. Only those patients who reported breathlessness “most days in the past month” were invited to complete the rest of the survey which included questions to identify patient and clinical characteristics and both clinical and informal support for their breathlessness. The modified Medical Research Council Dyspnoea Scale (mMRC) was used to assess the severity of exertion-related breathlessness. Questions related to both the participant’s current status and at the time they decided to present to the ED; demographic information, was breathlessness a reason for presentation, severity of breathlessness at decision to present and when completing the survey in the ED using a 4 point verbal scale (none, mild, moderate, severe), mMRC dyspnoea scale, duration of breathlessness (<6 m, 7 m to 2 yrs, >2 yrs), who was involved in the decision to present, who the patient discusses their breathlessness with and self-reported underlying diagnosis.

The data extracted from the clinical records of survey respondents who presented because of breathlessness included: demographic and clinical characteristics relating to the presentation; investigations/treatments provided for breathlessness; any documentation to indicate that the clinician had identified breathlessness as a cause of presentation (Any of the following-History: shortness of breath, SOB, dyspnoea. Observations: increased respiratory rate, tachypnea. Investigations: pulse oximetry, arterial blood gases, chest x-ray. Treatments in ED: inhaler, nebuliser, oxygen; co-morbidities and post-ED destination.)

### Clinical setting

The study was set in a tertiary teaching hospital serving a mixed urban/rural population with wide variation in affluence and deprivation. The ED is divided into major emergencies (majors) which receives patients the vast majority of whom arrive by ambulance and minor emergencies (minors) for “walk-in” patients. In response to clinical advice, the study was set in majors as it is the area most likely to receive patients with clinically significant breathlessness. In the event of a patient with clinically significant breathlessness self-reporting to minors, they would be re-directed to majors. In addition, by focusing on the majors area, the impact of acute-on chronic breathlessness on the ambulance service could be estimated. Ethics approval, including for the method of consent, was given by the NHS National Research Ethics Service Committee South Central-Hampshire B (Ref: 13/SC/0543) and institutional permission were obtained prior to data collection.

### Data collection

The patient-report survey was administered to consecutive adult attendees to majors from 7 am 12th May 2014 to 7 am 29th May 2014; the duration determined by the length of time to achieve the required sample size. Eligible participants were adults with capacity and sufficient clinical stability to complete the survey judged using routine clinical assessment by department staff. Completion was taken as implied consent. At the end of the survey, participants were invited to provide written consent for clinical record review. Clinical record data were extracted for consenting patients who had presented due to breathlessness.

### Sample size

Using a previous prevalence estimate of 3.2% [1] to estimate the prevalence to within one percentage point with a 95% confidence interval, a sample size of 1,191 for the prevalence survey was required.

### Statistical methods

The primary outcome measure was the prevalence of acute-on-chronic breathlessness as a cause for presentation to the ED. Descriptive statistics (proportion, mean, median, IQR, range) were used to present clinical and demographic data from the survey and clinical record review. Inferential statistics (Z test, Fisher’s Exact test and 95% confidence intervals) were used to calculate the primary outcome of prevalence and compare the sample with people presenting who were not breathless and also with the surrounding population. Analysis was undertaken on SPSS (Released 2011. IBM SPSS, Statistics for Windows, Version 20.0. Armonk, NY: IBM Corp).

## Results

### Prevalence of presentation to the ED by people with chronic breathlessness

Of the 1,212 presentations, 424 were made by people with chronic breathlessness; a prevalence of 35.0% (95% CI 32.2% to 37.7%).

### Study participants

**Fig. 1:**
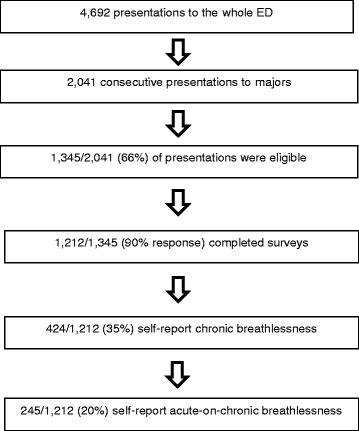
Flowchart of presentations to the ED during study period

The number of presentations in the study period is shown in the flowchart in Fig. 1.

The characteristics of the survey and clinical record review participants are summarized in Table 1. Breathlessness was among the reasons for presentation for 245 patients of whom 177 consented to clinical records review. The time between arriving at the ED and filling in the survey was approximately 30 min to one hour.

**Table 1:** Patient characteristics with respect to breathlessness and the presentation

Patient characteristics (self-report)	*n* = 245/1,212 (except as noted)
Age mean (SD)	65 yrs (19)
Gender	117 M (48%) 128 F (52%)
mMRC grade (*n* = 236)	
0	20 (8%)
1	28 (12%)
2	29 (12%)
3	72 (31%)
4	87 (37%)
Severity of breathlessness (*n* = 242) At decision to present	
None	5 (2%)
Mild	32 (13%)
Moderate	79 (33%)
Severe	126 (52%)
Whilst waiting in the ED	
None	33 (14%)
Mild	104 (43%)
Moderate	85 (35%)
Severe	21 (8%)
Duration of chronic breathlessness (*n* = 237)	
1 to 6 m	85 (36%)
7 m to 2 yrs	40 (17%)
More than 2 years	112 (47%)
Who does the patient talk to about their breathlessness?	
GP	178 (73%)
Practice nurse	14 (6%)
Respiratory nurse	34 (14%)
Breathing clinic	7 (3%)
Heart failure nurse	3 (1%)
Specialist doctor	29 (12%)
Macmillan nurse	5 (2%)
Long Term Conditions Nurse	16 (7%)
Family/friends	64 (26%)
Support group	1 (0.4%)
No one	34 (14%)
Factors relevant to ED presentation (self-report)	
Who was involved in the decision to present?	
Self	92 (37%)
*People known to patient*	
Family/friend	98 (40%)
Paid carer	9 (4%)
GP surgery	47 (19%)
Long Term Condition Nurse	4 (2%)
Heart failure nurse	0 (0%)
Respiratory nurse	4 (2%)
Macmillan nurse	3 (1%)
*Emergency services*	
Out of hours service	7 (3%)
NHS Direct	18 (7%)
999 (Emergency number)	14 (6%)
Paramedic	43 (18%)

### Characteristics

People with chronic breathlessness were older than those without (mean age with, 65[SD 19] vs without, 59 [SD 20]: mean difference 6.1 years; 95% CIs 3.7 to 8.6; *p* < 0.001), but there was no gender imbalance (45% men for both). The prevalence of presentation to majors by people with chronic breathlessness due to COPD [121/1,212 (10%; 8 to 12%)] or heart problems [165 (14%; 12 to 16%)] was higher than the prevalence of presentation to majors by people with chronic breathlessness due to asthma [54/1,212 (4.5%; 3.3 to 5.7%)] or by people with chronic breathlessness due to cancer [42/1,212 (3.5%; 2.7 to 4.3%)].

### Prevalence of presentation due to acute-on-chronic breathlessness

The prevalence of acute-on-chronic breathlessness was 20.2% (245/1,212, 95% CI 17.9% to 22.5%). Hospital activity records show that there were 4,692 presentations to both majors and minors during this period, therefore breathlessness sufficiently severe to necessitate an assessment in majors comprised *at least* 5.2% (245/4,692, 95% CI 4.6 to 5.9%) of all ED presentations.

### Presentations by participants with acute-on-chronic breathlessness (see Table 1)

People presenting with acute-on-chronic breathlessness reported significant levels of exertion-related breathlessness over the previous month (median mMRC 4, [interquartile range 3 to 5]) with two thirds (159/236) self-scoring mMRC grade 3 or 4. Nearly half (112/245) had experienced chronic breathlessness for more than 2 years.

When asked who they talked to about their breathlessness the most common practitioner consulted was their family doctor. A quarter said they talked to their family or friends and a significant minority (one in seven) said they didn’t talk to anyone at all about their breathlessness.

The median level of breathlessness at the time of survey completion was “mild”, reduced from “severe” at the time of decision to present. When asked who was involved in the decision to present that day just over a third said that they themselves, or family/friends were. The GP surgery or paramedic was involved in the decision in about one in five.

From case note review data (*n* = 177) approximately two thirds (121/177; 68%) of presentations were made outside of working hours defined as 8 am-6.30 pm Monday to Friday excluding public holidays [14]. Half (94/177; 53%) were re-attenders, having presented to the ED at least once in the 12 months before the index presentation.

“Breathing difficulties” was documented by the triage nurse as a primary presenting complaint in one third of people (56/177; 32%) and was the most common complaint documented (“illness” 27%; “chest pain” 23%; “other” 18%). Doctors documented difficulties with breathing (primary or one of the reasons) in two-thirds of case records (112/177; 63%).

Seven out of ten (122/177; 69%) of presentations due to acute-on-chronic breathlessness resulted in admission lasting on average 1 day (IQR 0 to 5; range 0 to 44). From hospital activity records during this period there were 1,615 hospital admissions from both majors and minors. Acute-on-chronic breathlessness was therefore a contributing factor in *at least* 7.6% (122/1,615, 95% CI; 6.3 to 8.9%) of all admissions from the ED. The proportion of presentations due to any cause to both majors and minors which resulted in admission in the survey time period was 34% (1,615/4,692).

## Discussion

### What did we find?

This study found that over one in three presentations to the majors area of the ED was by someone living with chronic breathlessness, and nearly one in five presentations were reported by the patient to be due to acute-on-chronic breathlessness. This is higher than the prevalence of chronic breathlessness in the general population (MRC Dyspnea scale grade ≥2, 8.9%) [15]. People presenting with chronic breathlessness had moderate to severe breathlessness at the decision to attend, and were twice as likely to be admitted to hospital as those presenting for other reasons. Most presented during “out-of-hours”.

### How does this compare to previous work?

This prevalence estimate of *at least* 5.2% from majors *and* minors is higher than previous ED reports [1, 3, 5]. The 9% reported by Langlo and colleagues, [4] like this study, excludes presentations to the minor injuries unit; much lower than the 20.2% reported here. However, previous studies used clinical record review rather than patient self-report; our study showed only two-thirds of study participants had any entry related to breathlessness in the clinical record. Although breathlessness at the time of the decision to present was rated by participants as “severe”, in the ED, this settled to “mild”. Therefore, by the time they were assessed by the clinician, they might have had no visible signs. Breathlessness may be “invisible” unless it is severe enough to be a clinical sign [16, 17].

Further, previous work noted the *primary* presenting complaint; in this study a third had “breathing difficulties” noted by the triage nurse, but the “chest pain” noted in others may have taken precedence as a reported primary reason even if they were breathless as well.

The prevalence of breathlessness as a reason to present to the ED is higher than that found for documented reason to attend the family practitioner (at least 5.2% *versus* approximately 1%) [18, 19]. However, if breathlessness was the “reason for encounter”, patients were 2.5 times more likely to be referred urgently to hospital by the family practitioner than those for whom breathlessness was not the “reason for encounter” [18].

The prevalence of hospital admissions for people attending the ED due to breathlessness was an estimated 7.6% of all admissions; lower than that found in other studies [5, 8]. This is likely to be an underestimate as patients who were too clinically unstable to complete the survey were excluded; a significant number of these may have had breathlessness, and be more likely to be admitted. In our study people presenting due to breathlessness were twice as likely to be admitted as others presenting to the ED for other reasons. This increased risk is consistent with previous findings [1, 5, 7, 9].

Most presentations by patients with chronic breathlessness were made by people with non-malignant cardio-respiratory disease. Although this is a single site study, the proportions of presentation by people with cardiorespiratory conditions are similar to the findings from the National Hospital Ambulatory Medical Care Survey and the AANZDEM observational study [5, 20]. However compared with the prevalence of such diseases in the community served by the hospital ED in this study, these are over-represented in the ED [21]. In contrast, the survey data regarding presentations by people with cancer was very similar to Quality Outcomes Framework [21] data relating to cancer in the local community. The reasons for this discrepancy are not clear, but it is interesting to reflect that the multi-disciplinary, cross-setting coordinated approach to the management of chronic non-malignant conditions in the UK has been slower to enter policy [22–24] and service delivery than for cancer care [25].

Three quarters of those presenting due to breathlessness scored grade 3 or above on the mMRC Dyspnea scale representing levels associated with significant activity limitation and negative consequences for well-being. Optimal care for such people should include quality management for both their underlying medical condition *and* their breathlessness, for which there are evidence-based interventions [26]. Although most participants say they discuss their breathlessness with their family practitioner few said they talked to specialist doctors, nurses or friends and family. It is surprising that few patients mentioned nurses as respiratory nurses, community matrons and long term conditions nurses have a liaison role and would be well-placed to help co-ordinate cross-setting care. Importantly, it identifies the family practitioner as a pivotal health professional with potential to co-ordinate optimal management [16]. In keeping with the pivotal role of the family doctor, two thirds of presentations to the ED occurred at times when their regular healthcare professional (family doctor, specialist nurse or physiotherapist) was not available. Some out-of-hours presentations such as those driven mainly by anxiety rather than serious exacerbation of the underlying pathology might be reduced if individual management plans included breathing crisis management, and training was given to both the patient and carer [10]. Healthcare professionals in the community available outside usual working hours also need to be skilled in the recognition and management of those with acute-on-chronic breathlessness episodes which could be managed in the community.

### Implications for clinical practice

It is important that ED clinicians assess a patient’s breathlessness routinely. Breathlessness is a stronger predictor of five year survival than tests of pulmonary function [27]. It is also associated with ED re-attendance and hospital admission and can therefore identify a group of people at higher risk for repeat presentation/admission. Knowledge of patients’ self-rated breathlessness can enable optimal care and symptom management; routine assessment of breathlessness in hospital is feasible [28]. Routine assessment of breathlessness in the ED may enable better management of patients both in the hospital and post discharge. Additionally approximately a third of presentations due to breathlessness resulted in discharge home from the ED. Some of these individuals may be those where anxiety and/or lack of self-management knowledge or skills is a significant factor. This issue can be challenging to discern during an episode of acute-on-chronic breathlessness, and may be better assessed in the community or clinic by the primary healthcare team. For these people presentation might have been avoided with optimised breathlessness care in the community and co-ordination of care between primary and secondary care [11].

### Strengths and limitations of this study

This survey was consecutive, including 24 h days and weekends, thus minimizing selection bias. There was a very high response rate (90%) and minimal missing data giving confidence that the sample was representative of those eligible to participate. However, the survey was administered during spring and a seasonal variation has been found by others, with the highest prevalence of breathlessness-related presentations during winter [5]. It was also conducted in a single site, however although there will inevitably be findings specific to this site, there are sufficient similarities to other published work to support their relevance. This study was performed in a city in the northeast of England which has a higher prevalence of COPD than the rest of England [21]. Owing to the wide variation in prevalence of these conditions nationally the study would need to be repeated before assuming generalizability. Furthermore, primary care is under-resourced in the study area; in the lowest quintile for number of family doctors per 100,000 of the population [29] and this may influence the number of presentations to the ED.

The survey was in English with no resources available for translation and therefore some patients may not have been able to take part. Only patients presenting to majors were surveyed and therefore a few patients who presented to minors but who were not re-directed to majors may have been missed. However, having used this method, we are able to comment on the impact on ambulance services. Further, patients who were too sick, or did not have mental capacity to complete the survey were excluded, and this group is likely to include people with breathlessness and those more likely to be admitted to hospital. Thus, if anything, our findings will be an underestimate of the total burden of acute-on-chronic breathlessness in the ED. Only 72% of the potential participants gave consent for clinical record review, which may have caused some selection bias in the clinical record data.

This study cannot determine whether presentation to the ED was appropriate or potentially avoidable. The assumption cannot be made that home discharge within a few hours equates to a preventable presentation. However, given other work to indicate that case-based complex interventions can reduce unscheduled hospital presentation for a variety of chronic medical conditions, [30] then further delineation and understanding of the needs of this patient group warrants further investigation.

## Conclusions

Acute-on-chronic breathlessness represents a significant burden for patients, their family carers and the emergency health services. There may be an important proportion of people whose breathlessness is not caused by a remediable exacerbation of underlying disease and who are discharged home within a few hours. The ED may not be the best place to coordinate the care of these patients, and further work to identify best management, for example, community-led breathlessness crisis plans, is warranted.
